# 4-Chloro-1*H*-pyrrolo­[2,3-*d*]pyrimidine

**DOI:** 10.1107/S1600536812034095

**Published:** 2012-08-08

**Authors:** Su-Lan Dong, Xiaochun Cheng

**Affiliations:** a College of Life Science and Chemical Engineering, Huaiyin Institute of Technology, Huaiyin 223003, Jiangsu, People’s Republic of China

## Abstract

The title compound, C_6_H_4_ClN_3_, is essentially planar with the pyrrole and pyrimidine rings inclined to one another by 0.79 (15)°. In the crystal, mol­ecules are connected *via* pairs of N—H⋯N hydrogen bonds, forming inversion dimers. These dimers are linked *via* C—H⋯N inter­actions, forming a two-dimensional network parallel to (10-1).

## Related literature

The title compound is an important organic inter­mediate in the synthesis of a drug which shows promising activity against HCV replication, see: Chang *et al.* (2010 ▶). For bond-length data, see: Allen *et al.* (1987 ▶).
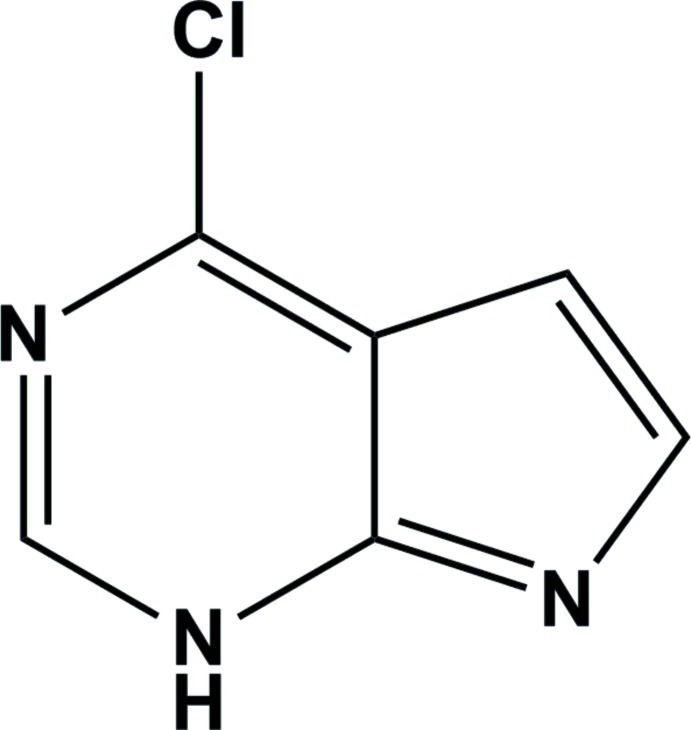


## Experimental

### 

#### Crystal data

C_6_H_4_ClN_3_
*M* *_r_* = 153.57
Monoclinic, 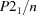

*a* = 10.8810 (19) Å
*b* = 5.2783 (9) Å
*c* = 12.751 (2) Å
β = 114.333 (3)°
*V* = 667.3 (2) Å^3^
*Z* = 4
Mo *K*α radiation
μ = 0.49 mm^−1^
*T* = 296 K
0.18 × 0.16 × 0.10 mm

#### Data collection

Enraf–Nonius CAD-4 diffractometer
Absorption correction: ψ scan (North *et al.*, 1968 ▶) *T*_min_ = 0.918, *T*_max_ = 0.953
3597 measured reflections
1273 independent reflections
1166 reflections with *I* > 2σ(*I*)
*R*_int_ = 0.017
3 standard reflections every 200 reflections intensity decay: 1%

#### Refinement

*R*[*F* ^2^ > 2σ(*F* ^2^)] = 0.052
*wR*(*F* ^2^) = 0.145
*S* = 1.00
1273 reflections
91 parameters
H-atom parameters constrained
Δρ_max_ = 0.58 e Å^−3^
Δρ_min_ = −0.43 e Å^−3^

### 

Data collection: *CAD-4 Software* (Enraf–Nonius, 1985 ▶); cell refinement: *CAD-4 Software*; data reduction: *XCAD4* (Harms & Wocadlo,1995 ▶); program(s) used to solve structure: *SHELXS97* (Sheldrick, 2008 ▶); program(s) used to refine structure: *SHELXL97* (Sheldrick, 2008 ▶); molecular graphics: *SHELXTL* (Sheldrick, 2008 ▶); software used to prepare material for publication: *SHELXTL*.

## Supplementary Material

Crystal structure: contains datablock(s) I, global. DOI: 10.1107/S1600536812034095/su2492sup1.cif

Structure factors: contains datablock(s) I. DOI: 10.1107/S1600536812034095/su2492Isup2.hkl

Supplementary material file. DOI: 10.1107/S1600536812034095/su2492Isup3.cml

Additional supplementary materials:  crystallographic information; 3D view; checkCIF report

## Figures and Tables

**Table 1:** Hydrogen-bond geometry (Å, °)

*D*—H⋯*A*	*D*—H	H⋯*A*	*D*⋯*A*	*D*—H⋯*A*
N2—H2⋯N1^i^	0.86	2.07	2.927 (3)	174
C6—H6⋯N3^ii^	0.93	2.57	3.315 (3)	137

Symmetry codes: (i) 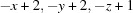; (ii) 
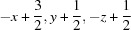
.

**Fig. 1.**
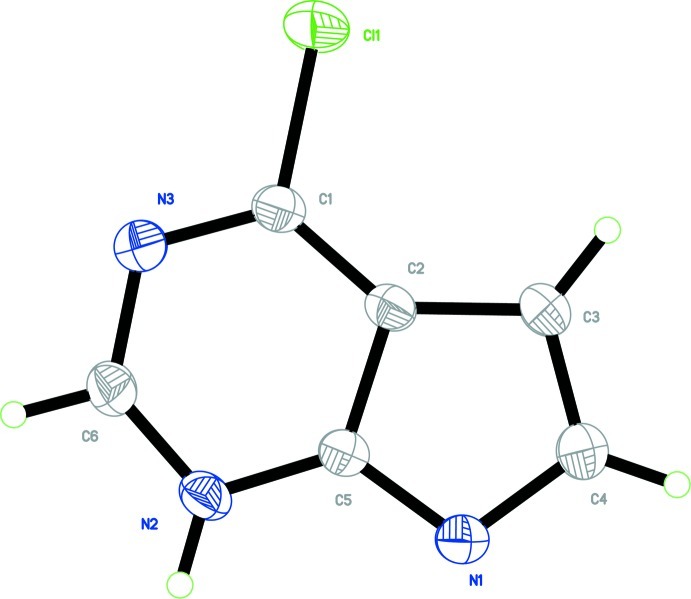
The molecular structure of the title molecule, with atom numbering. Displacement ellipsoids are drawn at the 50% probability level.

**Fig. 2.**
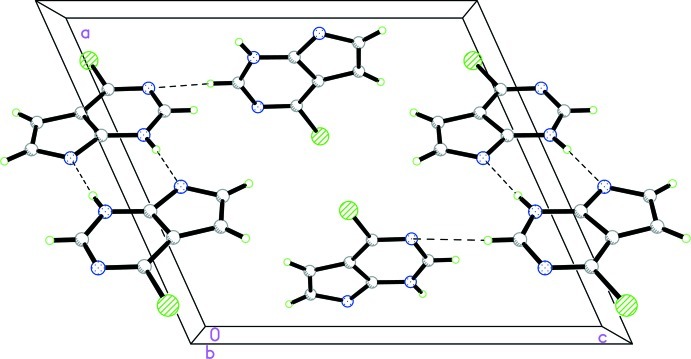
A view along the a axis of the crystal packing of the title compound. The N-H···N and C-H···N hydrogen bonds are shown as dashed lines (see Table 1 for details].

## supplementary crystallographic information

### Comment

The title compound is an important organic intermediate that has been used to synthesis a drug which has shown promising activity against HCV replication (Chang *et al.*, 2010).

The molecular structure of the title molecule is shown in Fig. 1. The bond lengths (Allen *et al.*, 1987) and angles are within normal ranges. The molecule is planar with the pyrrole ring (N1/C2-C5) and pyrimidine ring (N2/N3/C1/C2/C5/C6) being inclined to one another by only 0.79 (15)°.

In the crystal, molecules are connected via a pair of N-H···N hydrogen bonds to form inversion dimers, which are further linked via C-H···N interactions (Table 1 and Fig. 2). This results in the formation of a two-dimensional network parallel to (1 0 -1).

### Experimental

The title compound was prepared by a method reported in the literature (Chang *et al.*, 2010). A solution of phosphoryl trichloride (22.7 g, 158 mmol) in dichloromethane (50 ml) was added slowly to a solution of 3*H*-pyrrolo[2,3-*d*]pyrimidin-4(4aH)-one (10 g, 74 mmol). After being stirred for 6 h at reflux temperature, the solvent was filtered and the organic phase was evaporated on a rotary evaporator and gave the title compound. Colourless block-like crystals, suitable for X-ray diffraction analysis, were obtained by dissolving the solid (0.5 g, 3.26 mmol) in ethanol (25 ml) and evaporating the solvent slowly at room temperature for about 7d.

### Refinement

All the H atoms were positioned geometrically and constrained to ride on their parent: N-H = 0.86 Å, C—H = 0.93 Å with *U*_iso_(H) = 1.2U_eq_(N,C).

### Crystal data

**Table:**

C_6_H_4_ClN_3_	*F*(000) = 312
*M**_r_* = 153.57	*D*_x_ = 1.529 Mg m^−^^3^
Monoclinic, *P*2_1_/*n*	Mo *K*α radiation, λ = 0.71073 Å
Hall symbol: -P 2yn	Cell parameters from 4634 reflections
*a* = 10.8810 (19) Å	θ = 6.4–60.4°
*b* = 5.2783 (9) Å	µ = 0.49 mm^−^^1^
*c* = 12.751 (2) Å	*T* = 296 K
β = 114.333 (3)°	Block, colourless
*V* = 667.3 (2) Å^3^	0.18 × 0.16 × 0.10 mm
*Z* = 4	

### Data collection

**Table:**

Enraf–Nonius CAD-4 diffractometer	1166 reflections with *I* > 2σ(*I*)
Radiation source: fine-focus sealed tube	*R*_int_ = 0.017
Graphite monochromator	θ_max_ = 26.0°, θ_min_ = 3.2°
ω/2θ scans	*h* = −13→11
Absorption correction: ψ scan (North *et al.*, 1968)	*k* = −6→6
*T*_min_ = 0.918, *T*_max_ = 0.953	*l* = −14→15
3597 measured reflections	3 standard reflections every 200 reflections
1273 independent reflections	intensity decay: 1%

### Refinement

**Table:**

Refinement on *F*^2^	Primary atom site location: structure-invariant direct methods
Least-squares matrix: full	Secondary atom site location: difference Fourier map
*R*[*F*^2^ > 2σ(*F*^2^)] = 0.052	Hydrogen site location: inferred from neighbouring sites
*wR*(*F*^2^) = 0.145	H-atom parameters constrained
*S* = 1.00	*w* = 1/[σ^2^(*F*_o_^2^) + (0.0781*P*)^2^ + 0.6149*P*] where *P* = (*F*_o_^2^ + 2*F*_c_^2^)/3
1273 reflections	(Δ/σ)_max_ < 0.001
91 parameters	Δρ_max_ = 0.58 e Å^−^^3^
0 restraints	Δρ_min_ = −0.43 e Å^−^^3^

### Special details

**Table:**

Geometry. All e.s.d.'s (except the e.s.d. in the dihedral angle between two l.s. planes) are estimated using the full covariance matrix. The cell e.s.d.'s are taken into account individually in the estimation of e.s.d.'s in distances, angles and torsion angles; correlations between e.s.d.'s in cell parameters are only used when they are defined by crystal symmetry. An approximate (isotropic) treatment of cell e.s.d.'s is used for estimating e.s.d.'s involving l.s. planes.
Refinement. Refinement of *F*^2^ against ALL reflections. The weighted *R*-factor *wR* and goodness of fit *S* are based on *F*^2^, conventional *R*-factors *R* are based on *F*, with *F* set to zero for negative *F*^2^. The threshold expression of *F*^2^ > σ(*F*^2^) is used only for calculating *R*-factors(gt) *etc*. and is not relevant to the choice of reflections for refinement. *R*-factors based on *F*^2^ are statistically about twice as large as those based on *F*, and *R*- factors based on ALL data will be even larger.

### Fractional atomic coordinates and isotropic or equivalent isotropic displacement parameters (Å^2^)

**Table:**

	*x*	*y*	*z*	*U*_iso_*/*U*_eq_	
Cl1	0.62621 (8)	0.17642 (16)	0.50664 (6)	0.0659 (3)	
C1	0.7287 (2)	0.3943 (4)	0.4803 (2)	0.0401 (5)	
C2	0.8091 (2)	0.5538 (5)	0.56688 (18)	0.0385 (5)	
C3	0.8403 (3)	0.6051 (6)	0.6845 (2)	0.0533 (7)	
H3	0.8069	0.5213	0.7315	0.064*	
C4	0.9284 (3)	0.8009 (6)	0.7143 (2)	0.0581 (7)	
H4	0.9656	0.8744	0.7870	0.070*	
C5	0.8835 (2)	0.7273 (4)	0.53211 (19)	0.0365 (5)	
C6	0.8026 (3)	0.5695 (5)	0.3549 (2)	0.0448 (6)	
H6	0.8000	0.5698	0.2810	0.054*	
N1	0.9559 (2)	0.8772 (4)	0.62302 (18)	0.0470 (5)	
N2	0.8820 (2)	0.7376 (4)	0.42666 (16)	0.0411 (5)	
H2	0.9289	0.8447	0.4077	0.049*	
N3	0.7247 (2)	0.3972 (4)	0.37545 (17)	0.0461 (5)	

### Atomic displacement parameters (Å^2^)

**Table:**

	*U*^11^	*U*^22^	*U*^33^	*U*^12^	*U*^13^	*U*^23^
Cl1	0.0695 (5)	0.0685 (5)	0.0662 (5)	−0.0334 (4)	0.0343 (4)	−0.0032 (3)
C1	0.0408 (11)	0.0391 (11)	0.0427 (12)	−0.0039 (9)	0.0195 (9)	0.0030 (9)
C2	0.0379 (10)	0.0418 (12)	0.0393 (11)	−0.0043 (9)	0.0193 (9)	0.0025 (9)
C3	0.0585 (15)	0.0679 (17)	0.0406 (12)	−0.0180 (13)	0.0276 (11)	−0.0020 (12)
C4	0.0618 (16)	0.0768 (19)	0.0407 (13)	−0.0208 (14)	0.0261 (12)	−0.0115 (13)
C5	0.0357 (10)	0.0374 (11)	0.0386 (11)	−0.0018 (9)	0.0175 (9)	0.0019 (9)
C6	0.0557 (13)	0.0454 (13)	0.0373 (11)	−0.0035 (11)	0.0233 (10)	0.0020 (10)
N1	0.0476 (11)	0.0521 (12)	0.0452 (11)	−0.0138 (9)	0.0230 (9)	−0.0071 (9)
N2	0.0474 (11)	0.0397 (10)	0.0433 (10)	−0.0046 (8)	0.0260 (9)	0.0040 (8)
N3	0.0529 (12)	0.0441 (11)	0.0418 (10)	−0.0085 (9)	0.0201 (9)	−0.0025 (8)

### Geometric parameters (Å, º)

**Table:**

Cl1—C1	1.728 (2)	C4—H4	0.9300
C1—N3	1.319 (3)	C5—N2	1.339 (3)
C1—C2	1.378 (3)	C5—N1	1.356 (3)
C2—C5	1.409 (3)	C6—N2	1.312 (3)
C2—C3	1.421 (3)	C6—N3	1.341 (3)
C3—C4	1.353 (4)	C6—H6	0.9300
C3—H3	0.9300	N2—H2	0.8600
C4—N1	1.376 (3)		
			
N3—C1—C2	123.4 (2)	N2—C5—N1	126.6 (2)
N3—C1—Cl1	116.75 (18)	N2—C5—C2	124.9 (2)
C2—C1—Cl1	119.89 (17)	N1—C5—C2	108.5 (2)
C1—C2—C5	113.7 (2)	N2—C6—N3	127.6 (2)
C1—C2—C3	139.4 (2)	N2—C6—H6	116.2
C5—C2—C3	106.9 (2)	N3—C6—H6	116.2
C4—C3—C2	105.9 (2)	C5—N1—C4	107.4 (2)
C4—C3—H3	127.0	C6—N2—C5	113.9 (2)
C2—C3—H3	127.0	C6—N2—H2	123.0
C3—C4—N1	111.3 (2)	C5—N2—H2	123.0
C3—C4—H4	124.4	C1—N3—C6	116.5 (2)
N1—C4—H4	124.4		
			
N3—C1—C2—C5	2.1 (4)	C3—C2—C5—N1	−0.2 (3)
Cl1—C1—C2—C5	−177.86 (17)	N2—C5—N1—C4	−179.5 (3)
N3—C1—C2—C3	−179.7 (3)	C2—C5—N1—C4	0.0 (3)
Cl1—C1—C2—C3	0.3 (4)	C3—C4—N1—C5	0.1 (4)
C1—C2—C3—C4	−178.0 (3)	N3—C6—N2—C5	0.8 (4)
C5—C2—C3—C4	0.3 (3)	N1—C5—N2—C6	180.0 (2)
C2—C3—C4—N1	−0.2 (4)	C2—C5—N2—C6	0.5 (3)
C1—C2—C5—N2	−1.9 (3)	C2—C1—N3—C6	−1.1 (4)
C3—C2—C5—N2	179.4 (2)	Cl1—C1—N3—C6	178.90 (18)
C1—C2—C5—N1	178.6 (2)	N2—C6—N3—C1	−0.5 (4)

### Hydrogen-bond geometry (Å, º)

**Table:**

*D*—H···*A*	*D*—H	H···*A*	*D*···*A*	*D*—H···*A*
N2—H2···N1^i^	0.86	2.07	2.927 (3)	174
C6—H6···N3^ii^	0.93	2.57	3.315 (3)	137

Symmetry codes: (i) −*x*+2, −*y*+2, −*z*+1; (ii) −*x*+3/2, *y*+1/2, −*z*+1/2.
